# Dispersal Ability Determines the Role of Environmental, Spatial and Temporal Drivers of Metacommunity Structure

**DOI:** 10.1371/journal.pone.0111227

**Published:** 2014-10-23

**Authors:** André A. Padial, Fernanda Ceschin, Steven A. J. Declerck, Luc De Meester, Cláudia C. Bonecker, Fabio A. Lansac-Tôha, Liliana Rodrigues, Luzia C. Rodrigues, Sueli Train, Luiz F. M. Velho, Luis M. Bini

**Affiliations:** 1 Departamento de Botânica, Universidade Federal do Paraná, Curitiba, Paraná, Brazil; 2 Programa de Pós-graduação em Ecologia e Conservação, Universidade Federal do Paraná, Curitiba, Brazil; 3 Department of Aquatic Ecology, Netherlands Institute of Ecology (NIOO-KNAW), Wageningen, The Netherlands; 4 KU Leuven, University of Leuven, Laboratory of Aquatic Ecology, Evolution and Conservation, Leuven, Belgium; 5 Núcleo de Pesquisa em Limnologia, Ictiologia e Aqüicultura (Nupelia), Universidade Estadual de Maringá, Maringá, Brazil; 6 Departamento de Ecologia, Universidade Federal de Goiás, Goiânia, Brazil; McGill University, Canada

## Abstract

Recently, community ecologists are focusing on the relative importance of local environmental factors and proxies to dispersal limitation to explain spatial variation in community structure. Albeit less explored, temporal processes may also be important in explaining species composition variation in metacommunities occupying dynamic systems. We aimed to evaluate the relative role of environmental, spatial and temporal variables on the metacommunity structure of different organism groups in the Upper Paraná River floodplain (Brazil). We used data on macrophytes, fish, benthic macroinvertebrates, zooplankton, periphyton, and phytoplankton collected in up to 36 habitats during a total of eight sampling campaigns over two years. According to variation partitioning results, the importance of predictors varied among biological groups. Spatial predictors were particularly important for organisms with comparatively lower dispersal ability, such as aquatic macrophytes and fish. On the other hand, environmental predictors were particularly important for organisms with high dispersal ability, such as microalgae, indicating the importance of species sorting processes in shaping the community structure of these organisms. The importance of watercourse distances increased when spatial variables were the main predictors of metacommunity structure. The contribution of temporal predictors was low. Our results emphasize the strength of a trait-based analysis and of better defining spatial variables. More importantly, they supported the view that “all-or- nothing” interpretations on the mechanisms structuring metacommunities are rather the exception than the rule.

## Introduction

The identification of the mechanisms driving variation in and among local communities is central to community ecology. The role of environmental and spatial processes operating in multiple scales to shape local community composition is explicit in the metacommunity framework [1]–[5]. If community composition is mainly predicted by environmental variables, then niche-related mechanisms are considered the primary drivers of metacommunities and species are sorted across habitats [6], [7]. An alternative view has emphasized that the structure of local communities differ from each other mainly due to stochastic processes, including dispersal limitation and ecological drift [8]. In an effort to reveal the main mechanisms driving spatial variation in local communities, several studies have investigated the relative importance of environmental gradients and spatial processes in shaping metacommunity structure ([9], [10] and references therein). Not uncommonly, studies indicate that both niche and spatial processes may account for variation in community structure [11].

One may expect that the relative importance of deterministic (e.g., species sorting) and stochastic processes (e.g., dispersal) will be dependent on the dispersal ability of the biological groups under study. Recently, studies have compared organism groups with different dispersal abilities in the same set of habitats to test the hypotheses that: i) niche related processes are important in structuring local communities for organisms with high dispersal ability, and ii) spatial structure is a better predictor of local community composition for biological groups with low dispersal ability [9], [10], [12]. Organisms with high dispersal ability may be less affected by spatial structure simply because they reach suitable patches more often than those with low dispersal ability [13]. In this case, species are sorted according to their environmental requirements. In freshwater ecosystems, dispersal ability is generally inversely related to body size [9], [12], [14], [15]. Therefore, large-bodied organisms, such as fish and aquatic macrophytes, may have comparatively lower dispersal ability than small organisms, such as plankton and benthic invertebrates. This difference allows one to predict an increased role of environmental predictors in the structure of local communities from large to small organisms [9].

The structure of local communities also varies through time [16]. For instance, a recent study has found that temporal environmental variation is an important mechanism explaining zooplankton beta-diversity [17]. However, studies simultaneously testing the relative role of environmental, spatial and temporal processes on metacommunities are uncommon [18]. In this context, it is important to emphasize that in some ecosystems (e.g., floodplains), the magnitude of temporal variation in community structure may be as high or higher than the magnitude of spatial variation [19]. The test of this conjecture is increasingly relevant given the long list of environments changes caused by human activities [20].

For this study, we analyzed a dataset on different biological groups in the Upper Paraná River floodplain, Brazil. We tested the hypothesis that the relative role of environmental conditions in structuring local communities is high for communities composed by small organisms (with high dispersal ability). Conversely, the relative role of spatial variables in predicting community structure would increase for communities composed by large-bodied organisms. We also assessed the role of temporal dynamics on community structure in this highly dynamic system.

## Methods

### Study area

**Figure 1:**
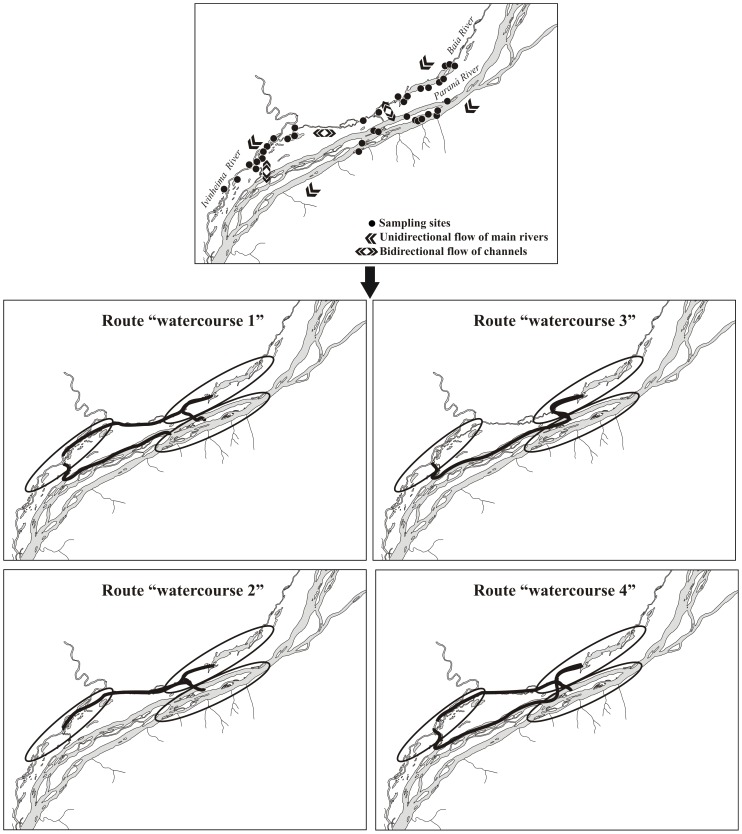
Possible routes of dispersion among sampling sites in the Upper Paraná River floodplain. These hypothetical routes were charted based on the unidirectional flow of main rivers and bidirectional flow of lateral channels.

The Upper Paraná River and its floodplain (Figure 1) represent the last unregulated stretch of the Paraná River in the Brazilian territory. It is an important area for several migratory fish species and still has high species diversity [21]. Sampling sites in the Upper Paraná River floodplain were located along an environmental gradient of limnological, hydrological, and biological variables [22] within a protected area called “APA das Ilhas e Várzeas do Rio Paraná”. All samplings were authorized by the Brazilian agency for environmental protection (Instituto Brasileiro do Meio Ambiente – IBAMA, https://www.ibama.gov.br).The hydrological regime is characterized by a dry season (June–September) and a wet season (October–February). However, due to hydrological control by recently built hydropower reservoirs, the frequency, amplitude, and duration of the floods have substantially changed [21].

### Sampling

We collected data on six biological communities: aquatic macrophytes, fish, benthic macroinvertebrates, zooplankton, periphyton, and phytoplankton. Sampling was carried out during February, May (wet season), August and November (dry season) in 2000 and 2001. Depending on sampling month, we sampled up to 36 sites spread throughout the Upper Paraná River floodplain (Figure 1). These sites included floodplain lakes permanently connected to rivers, floodplain lakes connected to rivers only during floods, and river channels.

We recorded the presence and absence of all aquatic macrophytes in the field from a boat, with the help of a grapnel to search for submerged vegetation. We determined fish abundance (individuals × 24 hours/1000 m^2^ gillnet) by standardized fishing using gill nets with different mesh sizes. We used a Petersen’s grab to collect benthic macroinvertebrates. The total number of individuals of each taxon per sample was used as the abundance data. We collected zooplankton samples by pumping 600 L of water through a 68 µm mesh net and, after laboratory procedures, data were expressed as individuals/m^3^. We sampled periphyton from petioles of *Eichhornia azurea* Kunth in the mature stage, as this macrophyte was common in most of the environments in the Upper Paraná River floodplain. Abundance was expressed in individuals/cm^2^. We used Van Dorn bottle to sample phytoplankton, and species densities were expressed as individual units (cells, coenobia, colonies, or filaments) per milliliter. Individuals of all biological groups were identified to the lowest taxonomic level possible. With the exception of benthic macroinvertebrates, identification reached species or genus level [22]. For benthic macroinvertebrates, some groups were identified as family, order or even class.

Although it is difficult to accurately classify organisms in terms of dispersal ability, we assumed, based on the body size and dispersal strategies [9], that microalgae (phytoplankton and periphyton) have the highest dispersal ability at the scale of the floodplain, whereas fish and macrophytes were expected to have the lowest. Zooplankton and macroinvertebrates were expected to have intermediate dispersal ability. Given the intricate spatial configuration of floodplain systems, with several dendritic watercourses temporarily or permanently connecting lakes, channels and main rivers, those groups dispersing directly through water may exhibit the lowest dispersal ability. Microorganisms may disperse via several vectors (e.g., air, watercourse, and attached to animals and plants).

We obtained the following environmental variables for each sampling site: depth (m), water temperature (°C), dissolved oxygen (mg/L), water transparency (m), pH, conductivity (µS/cm), total alkalinity (mEq/L), turbidity (NTU), total nitrogen concentration (µg/L), total phosphorus concentration (µg/L), chlorophyll-*a* (µg/L), total suspended matter (mg/L), and dissolved organic matter (mg/L). All environmental variables, except for pH, were log (*x*) transformed prior to the analyses described below. Details on sampling and laboratory procedures used to obtain the biological and environmental data can be found elsewhere [22].

### Data analysis

We used partial redundancy analysis (pRDA) to estimate the relative role of environmental, spatial and temporal predictors on the structure of the aquatic communities. As response matrices, we used abundance data for all biological groups (except for macrophytes because only presence/absence data are available for this group) and the total variance in the community data matrix was divided into unique and shared components of a set of environmental, spatial and temporal predictors [18]. As data are lacking for some groups in some sites and periods, we did not carry out an analysis with 36 sites and eight sampling periods for all biological groups (see Appendix S1).

The environmental matrix was composed of the limnological variables described above. We checked for collinearity among variables and removed variables that were strongly correlated with another variable before pRDA. Chlorophyll-*a* was not used as a predictor of periphyton and phytoplankton.

We used different strategies to generate spatial variables. Firstly, we calculated matrices of Euclidean (“overland”) and watercourses distances between sites (**D** and **W** respectively). Four possible scenarios of spatial relationships between the sites are possible considering the unidirectional flow of the main rivers (i.e., Paraná, Baia and Ivinheima) and bidirectional flows of lateral channels (see Figure 1). For instance, sampling sites located in the Baia River subsystem can be connected to sampling sites from the Ivinheima River subsystem by a lateral channel (Figure 1, left schemes) or by the Paraná River main channel (Figure 1, right schemes). Also, Ivinheima River sampling sites and Paraná River sampling sites can be connected through a lateral channel downstream (Figure 1, upper schemes) or upstream (Figure 1, lower schemes). We generated four matrices **W** to represent the possible organism dispersion routes. Spatial variables based on the five distance matrices described above (one **D** and four **W**) were created using Moran’s Eigenvector Maps [23]. Therefore, these spatial variables (i.e., eigenvectors extracted from the distance matrices) are different representations of how sampling sites are spatially related [24], [25]. We have also generated spatial predictors using asymmetric eigenvector maps (AEM) considering the directional flow of main rivers [26]. We selected only the eigenvectors with positive Moran’s *I* autocorrelation coefficients, assuming that these eigenvectors are proxies for dispersal processes or unmeasured environmental variables that are spatially structured.

The temporal matrix was composed by dummy variables differentiating sampling periods. For instance, a temporal matrix that had eight sampling periods was generated with seven vectors: each having “1” for a certain sampling period and “0” for the others. Thus, the temporal matrix was composed by dummy variables indicating that a group of sites was sampled at the same time. By using three explanatory matrices (environmental, spatial and temporal), eight variance components (or fractions of variation in canonical analysis; (see [27]) are generated in variation partitioning (see [18]): (1) Pure environmental, *E*: the fraction of variation in community structure explained by environmental variables that are neither spatially nor temporally structured; (2) Pure spatial, *S*: Spatial patterns in the species data that are independent of any temporal or environmental predictors included in the analysis; (3) Pure temporal, *T*: Temporal patterns in the species data that are independent of any spatial or environmental predictors included in the analysis; (4) *SE*: the variation in biological data explained by spatially structured environmental variables; (5) *TE*: the variation in biological data explained by temporally structured environmental variables; (6) *ST*: represents the explained variation that is co-structured in time and space, for instance in the case of temporally structured habitat connectivity; (7) *STE*: spatially and temporally structured environmental variation. This component, if important, indicates that the explanation of one predictor is correlated with the two others; (8) *U*: the unexplained variation in the community data - the fraction that cannot be explained by spatial, temporal or environmental predictors. These components were calculated using adjusted fractions, which take sample size and number of variables into account [27]. The significances of the fractions *E*, *S* and *T* were tested using 999 random permutations.

Before the analysis described above, presence-absence and abundance data were Hellinger transformed [27], [28]. Results were similar after excluding rare species (those occurring in only one sampling site). Therefore, analyses were done with the total dataset. We used the R language and environment for statistical computing [29] with ‘vegan’ [30] and ‘spdep’ [31] packages for analyses.

Following previous studies [4], [9], [32], we assumed that fish and aquatic macrophytes are, comparatively, poor dispersers and that macroinvertebrates, zooplankton, phytoplankton and periphyton generally have higher dispersal abilities. In addition, the fish dataset was divided into a table of sedentary fish and a table of migratory fish [33]. To create a coarse quantitative measure of dispersal ability, these biological groups were ranked in the following order: phytoplankton (1), periphyton (2), zooplankton (3), macroinvertebrates (4), migratory fish (5), sedentary fish (6) and aquatic macrophytes (7). We used Spearman rank correlation to test the relationship between this crude ranking of dispersal ability and the relative importance of environmental and spatial variables in predicting community structure (difference between components *E* and *S*). We recognize that the dispersal classification listed above is qualitative and not only reflects a general expected trend. It is, for instance, impossible to reliably assert that phytoplankton have higher dispersal than periphyton given both biological groups are comprised mainly by microalgae. Also, there is no doubt variation within groups. Yet, it remains that it is very likely that of all groups microalgae have the highest dispersal ability because of their abundances (sources of migrants) and small body size (see [9]). To take into account uncertainties in the way our measure of dispersal ability was created and thus increase robustness of our results, we repeated the Spearman rank test after considering different rank schemes (see Appendix S1).

## Results

**Figure 2:**
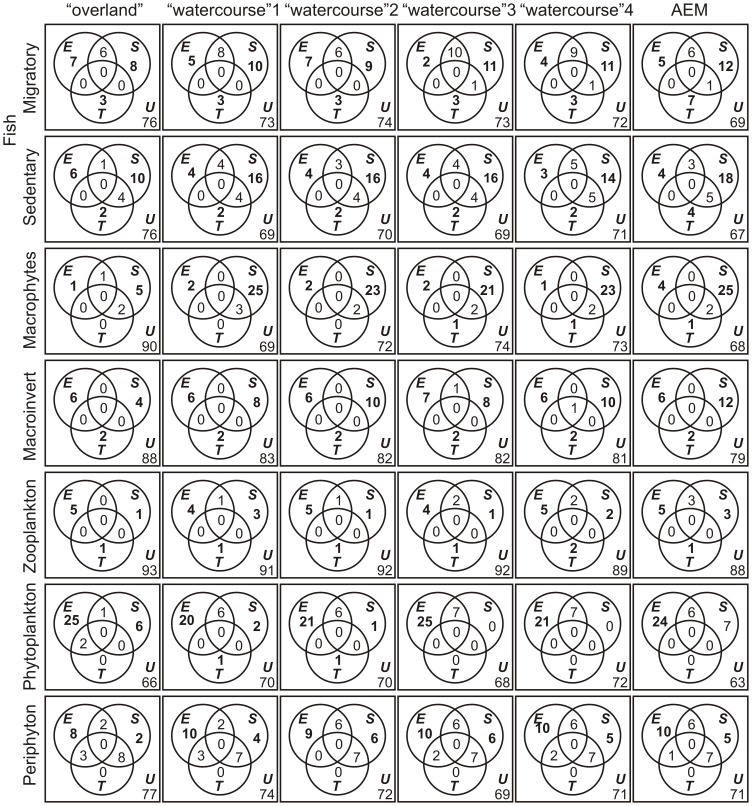
Results from partial redundancy analysis. Shown are the relative contributions (% of explanation) of environmental (*E*), spatial (*S*), and temporal (*T*) variables, as well as the shared components explaining variation in abundance of aquatic metacommunities (except for aquatic macrophytes, in which only presence/absence is available), using overland and four watercourse distances to generate spatial predictors. *U* = unexplained component. Zeros indicate values lower than 0.5%. The significance of the pure components (*E*, *S* and *T*) was tested using random permutations; bold numbers indicate significant values. Macroinvert = Benthic Macroinvertebrates.

Explanatory matrices explained up to 36.4% of the variation in biological datasets (Figure 2). Temporal variables (component *T*) significantly explained part of the variation in the structure of all groups except periphyton. The highest adjusted coefficient of determination associated with this fraction was obtained for migratory fish (6.7%). Spatial variables (component *S*) explained a significant proportion of the total variation in the structure of several groups. The lowest (and non-significant) component *S* was obtained for planktonic communities, independently of the type of distance used to create spatial variables. On the other hand, the *S* component was particularly high for aquatic macrophytes and sedentary fish when watercourse distances or AEM were used to generate spatial predictors. Environmental variables (component *E*) significantly accounted for part of the variation in the community structure of all groups. The highest shared fraction of variation was the spatially structured temporal variation (component *ST*), recorded for periphyton (Figure 2).

**Figure 3:**
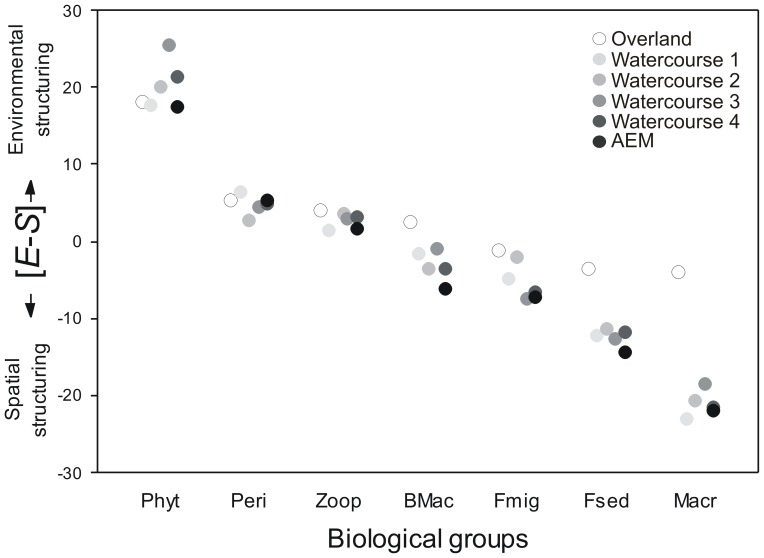
Difference in the contribution of environmental (*E*) and spatial variables (*S*) for the different biological groups, ranked according to the presumed dispersal ability. Different distance matrices were used to generate spatial predictors (one overland and four watercourse distances). *E* is the fraction of variation in community structure explained by environmental variables that are neither spatially nor temporally structured; *S* is the spatial patterns in the biological data that are independent of any temporal or environmental predictors. Phyt = phytoplankton; Peri = periphyton; Zoop = zooplankton; BMac = benthic macroinvertebrates; Fmig = migratory fish; Fsed = sedentary fish; Macr = macrophytes.

We found a negative correlation between the difference *E*–*S* and dispersal ability (Figure 3 and Appendix S1), indicating that variation in community structure of groups with high dispersal ability (e.g., phytoplankton) were better predicted by environmental variables. Conversely, spatial variables were the main predictors of variation in groups with lower dispersal ability (e.g., aquatic macrophytes). The relative roles of environmental and spatial variables in structuring periphyton, zooplankton and macroinvertebrate communities were intermediate compared to phytoplankton and macrophytes (Figure 3). These patterns were nearly independent of the type of distance matrix, i.e. the hypothesized dispersal routes used to generate the spatial variables, and of the dispersal ability ranks (e.g. whether phytoplankton or periphyton are considered to be the group with the highest dispersal ability; see abscissa of Figure 3 and Appendix S1).

## Discussion

Our results suggest that there exists an overall association in the study area, the Upper Paraná River floodplain, between the dispersal ability of different organism groups and the relative roles that species sorting and neutral spatial dynamics play in structuring their metacommunities. Our results are in line with the existing studies on this theme [4], [9], [10], [32], [34], which also found that the variation in community structure of organism groups with high dispersal ability is mainly accounted for by environmental variables, while spatial predictors are more important in groups with low dispersal ability [9], [10], [32], [35]. We assumed that macrophytes and fish, being the larger bodied-organisms in our study, have lower dispersal ability than the other taxa. Although fish actively search for habitats, dispersal in floodplains is not always evident given the complexity of channels and floodplain lakes and the fact that habitats can be temporarily isolated [19], [21]. We indeed observed that spatial variables were important in explaining variation in community structure for fish and macrophytes, and that the spatial scenario that takes watercourse connectivity into account had the highest explanatory power [25]. Environmental variables were especially important in structuring the local communities of periphyton and phytoplankton, small organisms with typically large population sizes and high dispersal abilities [14], [36]. In line with previous studies [4], [9], environmental drivers had a stronger contribution to explaining community structure in phytoplankton than in zooplankton. Although our results are in line with previous studies, to the best of our knowledge, only De Bie *et al*. [9] so far were able to formally test the relationship between the relative roles of processes driving metacommunity structure and dispersal ability inferred by body size. Other studies with similar goals (e.g., [32]) were not able to formally test this relationship due to the lower number of organism groups that differ in dispersal abilities that could be compared. To test for generality of this pattern, we encourage further studies to test this relationship using data on multiple biological groups that differ in dispersal abilities surveyed in the same set of sampling sites.

The role of dispersal processes cannot be inferred from the mere observation of a significant spatial component *S*. Variance component *S* may, for instance, also reflect the importance of unmeasured, spatially structured environmental variables [24], [37], [38]. Rather, it is a combination of results - significant component *S* combined with a negative relationship between the difference *E*–*S* and dispersal ability in a cross-group analysis - that constitutes strong evidence for the role of dispersal limitation. Although it is difficult to accurately rank the different study taxa with respect to their dispersal ability, we here work with a crude, robust ranking, with protists having the highest dispersal ability [39], and fish and macrophytes that generally need direct water connections having the lowest dispersal ability. As in earlier studies, zooplankton are expected to have lower dispersal ability than microalgae, but higher than fish [4], [9]. We recognize that macroinvertebrates could be split into flying dispersers in adult stage and those that have sedentary behavior (see [10], [34]). However, taxonomical resolution of our data did not allow us to accurately split benthic macroinvertebrate community into weak and strong dispersers. Even considering these caveats, the main pattern of an increase in the importance of spatial variables (relative to the importance of environmental factors) with decreasing dispersal ability proved to be robust to the choice of ranks used to create our measure of dispersal ability (see Appendix S1). Also, the comparison between sedentary and migratory fishes highlights the importance of a trait-based metacommunity analysis (see [9]) and provides further support for the interpretation of the variance component *S* as reflecting dispersal limitation.

Our results do not support that communities would be structured by only one of the four different metacommunity paradigms proposed by Leibold et al. [1]. Instead, our results suggest that both niche-driven and spatial processes contributed to a varying degree to the structure of the local communities and that this variation is structured by dispersal ability (see also [11]). Thus, although our results (i.e., high frequency of a significant component *E*) reflect generally strong species sorting [15], they also indicate that “all-or-nothing” interpretations on the mechanisms structuring metacommunities are rather the exception than the rule. The view of high variation in the importance of mechanisms underlying metacommunity structure has been supported by experimental [40], temporal [41] and spatial studies [9], [42], and our study adds to the evidence that this variation is in structuring mechanisms can be related to traits of the organisms [9]. In short, based on previous studies and on our results, we are of the opinion that, most likely, there is no “silver-bullet” explanation for metacommunity patterns.

Our results also highlight the relevance of better defining the spatial variables used in variation partitioning analysis [4], [25], [26], [35], [43]. For instance, when watercourse distances or AEM were used instead of overland distances, we recorded a substantial increase in the magnitude of component *S* for sedentary fish (from 9.9% to an average of 16.1%) and macrophytes (from 5.4% to an average of 23.4%; see Figure 2). This reflects that direct hydrological connections are important for dispersal of many organisms that live in floodplain river systems, particularly fish and macrophytes. Surprisingly, AEM, accounting for directional flow of the main rivers [26], did not generate better spatial predictors than symmetric eigenvector maps (see Figures 2 and 3). A likely explanation is that some habitats are connected by channels that exhibit bidirectional flow (see Figure 1). In short, our results reinforce that, at least in floodplain systems and river networks, ecologists interested in quantifying the relative role of spatial and environmental variables on community structure should go beyond the evaluation of simplistic spatial proxies [26], [35], [44], [45].

Floodplains are fundamentally seasonal systems, mainly due to the pervasive effects of the floods [46]. Indeed, studies carried out worldwide [47], [48] and in the Upper Paraná River itself [49], [50], indicate that floods account for important ecological patterns (e.g., species distribution) and processes (e.g., primary productivity and decomposition rates) in these systems. As a result, one could envisage that the role of spatial variables in explaining variation in community structure would be dependent on temporal predictors. It can, for instance, be expected that during high water periods, when there is a high level of connectivity within the floodplain, the role of spatial variables should be decreased. In this context, the lack of a strong temporal signal was a surprising result. Our data suggest that temporal dynamics do not massively change metacommunity structure, even in a floodplain setting, where the flood pulse is known to be a major structuring factor ([19], [46] and references therein). Yet, we need to be cautious in generalizing our result, as the flood pulse in the study system is reduced because of the construction of dams [21]. Also, our study involved only two years of sampling, and thus may underestimate long-term effects. Irrespective of these caveats, we found that temporal predictors (component *T*) were generally low, and highest (up to 6.7%) for those groups were expected to show much temporal variability, such as migratory fish.

We tried to circumvent at least some of the caveats that have commonly been discussed in the metacommunity literature, such as, for instance, the limitations of snapshot sampling [4]. Moreover, the combination of a trait-based analysis (here through comparing patterns across different organism groups along a body size gradient and by comparing sedentary and migratory fish) and the inclusion of better spatial variables, allowed us to be more confident in the interpretation of the spatial variation component as reflecting the role of dispersal ability [9], [24], [37]. Yet, we cannot discard the possibility that relevant environmental predictors were missing in our dataset [38]. We did not, for instance, include sediment characteristics, which might be important for macroinvertebrates. In addition, long-term ecological studies are necessary to properly evaluate the explanatory power of temporal processes on shaping metacommunities.

In conclusion, by analyzing data on different biological groups, we supported the hypothesis that the relative role of environmental and spatial processes on structuring local communities depends on the dispersal ability of these organisms [4], [9], [10]. We also demonstrated that spatial variables generated using watercourse distances and AEM (see also [4], [25], [26]) resulted in better estimates of the spatial drivers than geographical distances particularly when spatial structuring is the main mechanism in metacommunities. This is in line with a growing number of studies showing that one need to model connectedness of river networks. Finally, our study adds to the evidence that trait-based analyses [9] provide a deeper understanding of processes underlying metacommunity structure.

## Supporting Information

Appendix S1 (DOCX)
