# Supplementary material for: Dispersal Ability Determines the Role of Environmental, Spatial and Temporal Drivers of Metacommunity Structure
Source: PLoS One. 2014 Oct 23;9(10):e111227. doi: 10.1371/journal.pone.0111227 (PMC4207762; doi:10.1371/journal.pone.0111227)
Supplement: Appendix S1 — (DOCX) [file pone.0111227.s001.docx]

**Supporting Information**

**Appendix S1**

**Table 1.** List of available data for variation partitioning.

| **Community** | **February** | **May** | **September** | **November** | **Number of sampling sites** |
| --- | --- | --- | --- | --- | --- |
|  | **2000/2001** | **2000/2001** | **2000/2001** | **2000/2001** |  |
| Macrophytes | 🗸/🗸 | 🗸/🗸 | 🗸/🗸 | 🗸/🗸 | 36 |
| Fish | 🗸/🗸 | 🗸/🗸 | 🗸/🗸 | 🗸/🗸 | 20 |
| Benthic macroinvertebrates | 🗸/🗸 | 🗸/🗸 | 🗸/🗸 | 🗸/🗸 | 36 |
| Zooplankton | 🗸/🗸 | 🗸/🗸 | 🗸/🗸 | 🗸/🗸 | 36 |
| Periphyton | 🗸/🗸 | 🗸/🗸 | 🗸/🗸 | 🗸/🗸 | 32 |
| Phytoplankton | 🗸/🗸 | 🗸/🗸 | 🗸/🗸 | 🗸/🗸 | 30 |

*Dispersion limitation analysis*

We initially proposed the following rank of dispersal ability (from high to low ability): phytoplankton (1), periphyton (2), zooplankton (3), macroinvertebrates (4), migratory fish (5), sedentary fish (6) and aquatic macrophytes (7). This rank is based on the assumption that, in aquatic ecosystems, dispersal ability tends to be negatively related to body size for scales similar as considered in this study (see main text). To take into account uncertainties in the how we rank dispersal ability (or limitation) , we repeated the Spearman rank test after considering different rank schemes as a sensitivity analysis. The first rank scheme (A) was the one depicted in the abscissa of Figure 3 and explained in the main text. A second Spearman rank correlation was tested between *E*-*S* and a new dispersal variable (following scheme B) after switching the ranks of periphyton (rank = 1) and phytoplankton (2) and maintaining the other ranks as in the scheme A. The third Spearman rank correlation was estimated after switching the ranks of macroinvertebrates (3) and zooplankton (4), generating scheme C. The fourth Spearman rank correlation was estimated after changing the ranks of sedentary (5) and migratory fish (6), generating scheme D. The last Spearman rank correlation was estimated between *E*-*S* and a dispersal limitation measure that considered the rank of macrophytes equal to six and the one of sedentary fish equal to seven (scheme E). Thus, the rank schemes used to create our measure of dispersal limitation (1=low, 2, 3, 4, 5, 6, 7 =high) were the following (changes in relation to scheme A are highlighted in red):

| Group | Schemes | | | | |
| --- | --- | --- | --- | --- | --- |
|  | A | B | C | D | E |
| Phytoplankton | 1 | 2 | 1 | 1 | 1 |
| Periphyton | 2 | 1 | 2 | 2 | 2 |
| Zooplankton | 3 | 3 | 4 | 3 | 3 |
| Macroinvertebrate | 4 | 4 | 3 | 4 | 4 |
| Migratory fish | 5 | 5 | 5 | 6 | 5 |
| Sedentary fish | 6 | 6 | 6 | 5 | 7 |
| Aquatic Macrophyte | 7 | 7 | 7 | 7 | 6 |

The results of the Spearman rank correlation between *E-S* and our ranking scenarios with respect to dispersal ability, considering the different distances used to create spatial variables, were:

| Distance | Comparison | Spearman's *r* | *P* |
| --- | --- | --- | --- |
| Overland | *E* - *S* rank A | -1.00 |  |
|  | *E* - *S* rank B | -0.96 | 0.000 |
|  | *E* - *S* rank C | -0.96 | 0.000 |
|  | *E* - *S* rank D | -0.96 | 0.000 |
|  | *E* - *S* rank E | -0.96 | 0.000 |
|  |  |  |  |
| **W**_1_ | *E* - *S* rank A | -1.00 |  |
|  | *E* - *S* rank B | -0.96 | 0.000 |
|  | *E* - *S* rank C | -0.96 | 0.000 |
|  | *E* - *S* rank D | -0.96 | 0.000 |
|  | *E* - *S* rank E | -0.96 | 0.000 |
|  |  |  |  |
| **W**_2_ | *E* - *S* rank A | -0.93 | 0.025 |
|  | *E* - *S* rank B | -0.86 | 0.014 |
|  | *E* - *S* rank C | -0.82 | 0.023 |
|  | *E* - *S* rank D | -0.86 | 0.014 |
|  | *E* - *S* rank E | -0.89 | 0.007 |
|  |  |  |  |
| **W**_3_ | *E* - *S* rank A | -1.00 |  |
|  | *E* - *S* rank B | -0.96 | 0.000 |
|  | *E* - *S* rank C | -0.96 | 0.000 |
|  | *E* - *S* rank D | -0.96 | 0.000 |
|  | *E* - *S* rank E | -0.96 | 0.000 |
|  |  |  |  |
| **W**_4_ | *E* - *S* rank A | -1.00 |  |
|  | *E* - *S* rank B | -0.96 | 0.000 |
|  | *E* - *S* rank C | -0.96 | 0.000 |
|  | *E* - *S* rank D | -0.96 | 0.000 |
|  | *E* - *S* rank E | -0.96 | 0.000 |
|  |  |  |  |
| **AEM** | *E* - *S* rank A | -1.00 |  |
|  | *E* - *S* rank B | -0.96 | 0.000 |
|  | *E* - *S* rank C | -0.96 | 0.000 |
|  | *E* - *S* rank D | -0.96 | 0.000 |
|  | *E* - *S* rank E | -0.96 | 0.000 |
